# From incision to empowerment: Enhancing maternal recovery and self-care through structured post-caesarean support

**DOI:** 10.6026/973206300212744

**Published:** 2025-08-31

**Authors:** Jency Indira Kuppan, Sumathi Chandran, Shankar Shanmugam Rajendran, Vanitha Narayanasamy, Kavitha Ethiraj, Chithra Gopalan, Azhgar Jan Thoulath Khan

**Affiliations:** 1 Department of Obstetric and Gynecological Nursing, College of Nursing, Madras Medical College, The Tamil Nadu, Dr.MGR Medical University, Chennai, Tamil Nadu, India; 2 Department of Obstetrics and Gynecology Nursing, Institute of Obstetrics and Gynecology, Government Hospital for Women and Children, Madras Medical College, The Tamil Nadu, Dr.MGR Medical University, Chennai, Tamil Nadu, India; 3 Department of Child Health Nursing, College of Nursing, Madras Medical College, The Tamil Nadu, Dr.MGR Medical University, Chennai, Tamil Nadu, India; 4 Department of Community Health Nursing, College of Nursing, Madras Medical College, The Tamil Nadu, Dr.MGR Medical University, Chennai, Tamil Nadu, India; 5 Department of Medical Surging Nursing, College of Nursing, Madras Medical College, The Tamil Nadu, Dr.MGR Medical University, Chennai, Tamil Nadu, India

**Keywords:** Caesarean section, post-operative care, self-care, recovery, maternal perception, supportive nursing intervention, mixed-method study

## Abstract

The effect of a supportive post-operative program for improving recovery and self-care in post-caesarean mothers is of interest. The program, including pain management, wound care, nutrition support and emotional counseling, was shown to significantly enhance recovery compared to a control group. Quantitative results revealed improvements in pain reduction, mobility and self-care confidence. Thus, we show the emotional impact of LSCS and the importance of family support. The study underscores the value of structured nursing interventions for promoting holistic maternal recovery.

## Background:

A Lower Segment Caesarean Section (LSCS) is a common surgical procedure that helps ensure the safe delivery of babies when normal childbirth becomes risky [1]. However, for many mothers, the journey does not end in the operation theatre. After a caesarean, women often experience pain, fatigue, restricted movements and emotional struggles that affect their ability to care for themselves and their newborn [2]. In India, especially in urban areas, the number of C-sections is rising steadily [3]. Despite this, many mothers are discharged with minimal guidance on recovery, self-care, or coping with the physical and emotional challenges that follow [4]. Simple activities like getting out of bed, breastfeeding, or changing position become difficult in the early post-operative days [5]. Lack of proper instruction and emotional support leads many women to depend heavily on their family members, increasing caregiver burden and lowering the mother's confidence [6]. Traditional care focuses on wound checks and medicines, but often fails to address the deeper need for structured support, education and psychological reassurance during this sensitive period. Supportive post-operative nursing interventions can help bridge this gap by offering step-by-step guidance on self-care, wound management, pain control, early walking and emotional adjustment [7]. These strategies aim not just to heal the body, but also to strengthen the mother's ability to care for herself and her baby with confidence [8]. This study was inspired by real-life clinical experiences, where the researcher observed post-caesarean mothers feeling lost, anxious and unprepared for recovery after discharge. Therefore, it is of interest to determine the nurse-led supportive program could improve recovery, boost self-care practices and enhance the overall well-being of mothers who have undergone LSCS.

## Methodology:

The study aimed to evaluate the effectiveness of a supportive post-operative program on recovery, self-care and maternal perception of caesarean section at a selected tertiary care hospital in Chennai. An explanatory mixed-method design with a quasi-experimental approach was used. The research was conducted in the post-operative wards of IOG, Chennai, after obtaining ethical clearance and official permissions. In the quantitative phase, 60 post-caesarean mothers were selected using convenience sampling and divided into two groups: experimental (n = 30) and control (n = 30). The experimental group received a five-day supportive program, which included pain management, wound care, early ambulation, breastfeeding support, dietary advice and emotional guidance. The control group received routine postnatal care. The effectiveness of the intervention was measured using pre- and post-tests, including the Obstetrics Quality of Recovery Scale and the Self-Care Assessment Tool. Data were analyzed using SPSS with descriptive and inferential statistics, considering a significance level of p ≤ 0.05. In the qualitative phase, five mothers were selected through purposive sampling. Semi-structured interviews were conducted to explore the emotional, cultural and personal aspects of recovery and maternal perception of caesarean birth. The data from these interviews were analyzed through thematic analysis to gain insights into the mothers' experiences and perceptions.

## Results:

Most of the post-caesarean mothers were between 21-30 years (46.67%) and 65% were primigravida. More than half had only secondary education and many were low-income homemakers. Both groups were similar at the beginning, with no significant differences in their background details. From the qualitative data, six major themes were identified: fear about surgery, physical discomfort, difficulties in baby care, lack of self-care knowledge, emotional need for support and trust in nurses. Initially, mothers felt anxious and confused, but after receiving proper nursing guidance, they felt more confident and emotionally supported. In the quantitative phase, recovery scores were low in both groups during the pre-test. After the intervention, 70% of mothers in the experimental group reached high recovery levels, while 76.67% of mothers in the control group stayed at low levels. The improvement of 61.5 points in the experimental group was statistically significant (P < 0.001, Table 1 - see PDF). Similarly, self-care scores were initially low in both groups. After the program, 66.67% of mothers in the experimental group showed moderate to high self-care ability, but 83.33% of the control group remained low. The experimental group showed a significant improvement of 56.2 points (P < 0.001, Table 2 - see PDF). There was a moderate negative correlation (r = -0.42, P = 0.001) between self-care and recovery scores, meaning as self-care improved, recovery also increased. This correlation was significant only in the experimental group. Younger mothers (21-25 years), those with normal BMI and primigravida women showed better results and these associations were statistically significant only in the experimental group.

## Discussion:

This study demonstrated that a supportive post-operative program facilitated faster recovery and improved self-care management among post-caesarean mothers. These findings are supported by Mdoe *et al.* (2024) [9] who also reported that structured nursing care improves postnatal recovery and promotes independence. The positive correlation between self-care and recovery echoes Tazreean *et al.* (2022) [10], highlighting that early mobility and proper guidance speed up healing. Qualitative findings in this study, like emotional reassurance and trust in nurses, align with Tinmaz *et al.* [11] who emphasised the emotional needs of LSCS mothers and the value of nurse-led support. Overall, this study strongly supports integrating nurse-led, patient-centred care into routine post-caesarean recovery to enhance both physical and emotional well-being.

## Conclusion:

Post-operative nursing interventions significantly improved recovery and self-care among post-caesarean mothers. Thus, we demonstrate that emotional needs and nurse support are key factors in enhancing maternal confidence and well-being.
